# Exploring the Clinical Utility of Pancreatic Cancer Circulating Tumor Cells

**DOI:** 10.3390/ijms23031671

**Published:** 2022-01-31

**Authors:** Dannel Yeo, Althea Bastian, Heidi Strauss, Payal Saxena, Peter Grimison, John E. J. Rasko

**Affiliations:** 1 Li Ka Shing Cell & Gene Therapy Program, The University of Sydney, Camperdown, NSW 2050, Australia; dannel.yeo@sydney.edu.au (D.Y.); althea.bastian@sydney.edu.au (A.B.); heidi.strauss@sydney.edu.au (H.S.); 2 Faculty of Medicine and Health, The University of Sydney, Camperdown, NSW 2050, Australia; payal.saxena@sydney.edu.au (P.S.); peter.grimison@lh.org.au (P.G.); 3 Cell and Molecular Therapies, Royal Prince Alfred Hospital, Sydney Local Health District (SLHD), Camperdown, NSW 2050, Australia; 4 Division of Gastroenterology, Department of Medicine, Royal Prince Alfred Hospital, Sydney Local Health District, Camperdown, NSW 2050, Australia; 5 Medical Oncology, Chris O’Brien Lifehouse, Camperdown, NSW 2050, Australia; 6 Gene and Stem Cell Therapy Program, Centenary Institute, The University of Sydney, Camperdown, NSW 2050, Australia

**Keywords:** liquid biopsy, biomarker, prognosis, screening, stem cell, organoids, precision medicine, personalized medicine

## Abstract

Pancreatic ductal adenocarcinoma (PDAC) is the most frequent pancreatic cancer type, characterized by a dismal prognosis due to late diagnosis, frequent metastases, and limited therapeutic response to standard chemotherapy. Circulating tumor cells (CTCs) are a rare subset of tumor cells found in the blood of cancer patients. CTCs has the potential utility for screening, early and definitive diagnosis, prognostic and predictive assessment, and offers the potential for personalized management. However, a gold-standard CTC detection and enrichment method remains elusive, hindering comprehensive comparisons between studies. In this review, we summarize data regarding the utility of CTCs at different stages of PDAC from early to metastatic disease and discuss the molecular profiling and culture of CTCs. The characterization of CTCs brings us closer to defining the specific CTC subpopulation responsible for metastasis with the potential to uncover new therapies and more effective management options for PDAC.

## 1. Introduction

Over recent decades, advances in cancer detection, monitoring, and management have dramatically improved the survival rates and quality of life in cancer patients. Unfortunately, pancreatic cancer has not shared these improvements with the 5-year survival rate remaining below 10% [1]. In 2021, it is estimated that there will be over 60,000 new cases and over 48,000 deaths making it the fourth most common cause of cancer-related deaths in the United States of America [2]. Based on current trends, it is expected to become the second leading cause of cancer-related death by 2030 [3].

Pancreatic ductal adenocarcinoma (PDAC) accounts for over 90% of all pancreatic cancers. Many factors contribute to PDAC’s high mortality rate and poor prognosis. PDAC is generally asymptomatic in its early stages resulting in the majority of patients diagnosed with late-stage disease. Surgical resection remains the only potentially curative treatment option for PDAC patients, however less than 20% are suitable at diagnosis due to metastatic disease [4]. Even amongst suitable recipients, the 1-year survival rate following surgery is 20% with almost 80% of these patients developing recurrence [5]. Systemic chemotherapy (FOLFIRINOX: combination of folinic acid, fluorouracil (5-FU), irinotecan, and oxaliplatin; or combination of gemcitabine and nab-paclitaxel) is commonly used as first-line treatment for metastatic disease [6,7]. However, response rates and survival improvements are limited, and chemotherapy-related toxicities reduce their wider utility [8,9]. Although CA19-9 (carbohydrate antigen 19-9) is widely used as a serological marker in monitoring PDAC progression and response as a tumor burden marker, it is only elevated in 80% of cases and does not assist in predicting progression or response [10]. Hence, there is a need for superior biomarkers which can help stratify patients based on their risk of progression, recurrence and chemotherapy response, thereby enabling optimal and effective treatment management for patients.

## 2. Liquid Biopsies in PDAC

Tissue sampling for diagnosis is commonly carried out by endoscopic ultrasound (EUS)-guided biopsy [11,12]. Percutaneous radiologically-guided biopsy and open surgical biopsy are other biopsy procedures but are generally uncommon. EUS-guided biopsy requires anesthesia and carries a small risk of adverse events, so is therefore not suitable for repeated sampling [13,14]. Additionally, despite the high sensitivity of EUS-guided biopsies, the heterogeneity of tumor tissue and presence of abundant tumor stroma can impair the negative predictive value which can range from 40–70% [11,15,16]. 

In contrast to tissue biopsies, sampling peripheral blood is a simple, minimally invasive routine procedure. The detection of biomarkers within the peripheral blood, termed “liquid biopsy”, has been increasingly examined for a range of potential applications spanning cancer diagnosis to treatment [17]. The ease and minimal risk of peripheral blood collection allows for multiple samples to be collected from a patient, enabling longitudinal, long-term disease surveillance and monitoring. However, in order to fully utilize the potential of liquid biopsies, reliable blood-based biomarkers must first be identified and validated.

To date, CA19-9 and CEA (carcinoembryonic antigen) are the only routinely used clinical blood-based biomarkers for PDAC, with high levels indicative of advanced tumor stage [18,19]. While CA19-9 and CEA both have high specificity in PDAC (0.82 and 0.73, respectively) their sensitivity is limited (0.43 and 0.78, respectively) [19]. CA19-9 also has a number of caveats regarding its use, including being also elevated in benign disease and not being expressed in up to 10% of the Caucasian population who do not express the Lewis antigen [20]. Due to the potential of liquid biopsy biomarkers, efforts to identify other potential blood-based biomarkers alone or in conjunction with CA19-9/CEA have been undertaken. These include circulating tumor DNA (ctDNA) [21,22], exosomes [23], metabolites [24], non-coding RNA [25], and circulating tumor cells (CTCs) [26,27]. These biomarkers each have their own advantages and disadvantages in their application as a cancer diagnostic, prognostic and predictive biomarker. 

Circulating tumor DNA (ctDNA) represents a subset of cell-free DNA (cfDNA). Marked variations in the amount of cfDNA has been detected in healthy individuals and is associated with a range of conditions such as inflammation and tissue damage during intense physical activity [28,29]. ctDNA has a short half-life which can result in low yield and susceptibility to degradation during processing or inappropriate sample handling [17,30]. Sensitivity for KRAS-specific ctDNA has been reported in 27% to 81% of PDAC patients and the identification of targetable mutations is generally limited to patients with high ctDNA levels [30,31]. Thus far, ctDNA detection has been shown to predict PDAC recurrence, presence of minimal residual disease, and correlates with patient survival [32,33,34]. 

Exosomes are small extracellular vesicles with sizes between 30–150 nm that are secreted by all cell types and are responsible for intercell communication [35]. Although research into cancer exosomes commenced relatively recently, there has been much excitement. Similar to cfDNA, sensitivity in cancer-specific exosomes has been reported to range widely in PDAC patients, where 50% to 100% has been reported [23]. Exosomal markers have been identified for PDAC diagnosis, recurrence, and prognosis including miR-21, miR-451, miR-196a, miR-1246, and glypican-1 [36]. Isolation methods for exosomes, such as ultracentrifugation are currently inefficient and inconsistent, leading to difficulties in their characterization [37]. However, their clinical applicability as a biomarker in PDAC will continue to grow as well as their therapeutic applications as a delivery system [38,39].

CTCs are a rare subset of tumor cells found in the blood of solid tumor patients, functioning as the “seeds” of metastasis. They offer potential advantages over competing liquid biopsy biomarkers, particularly the ability to undertake downstream functional characterization. CTCs provide multiple opportunities to determine their phenotypic origins by way of surface-antigen identification and single-cell genomic and gene expression analysis [40,41]. The isolation of viable CTCs provides the added advantage of allowing further culturing and characterization of live cells by ex vivo culture or animal xenografts, providing a deeper understanding of an individual’s tumor characteristics [42,43]. This capacity goes beyond solely prognostic and diagnostic purposes and may help direct targeted, personalized treatments. This is of particular significance in PDAC where there are limited treatments available and a targeted approach may improve outcomes. Targeted therapy in PDAC has been shown to result in longer median survival where 26% of PDAC patients were found to harbor an actionable genomic alteration [44,45]. While genomic and transcriptomic analysis of tissue biopsy samples have been undertaken, there are limitations in the quantity of material obtained, heterogeneity of tumor cells, and contamination by stromal cells and other tissues. However, it is still unknown how representative CTCs are (this will be discussed in more detail in the “CTC Characterization” section).

CTCs may be a more accessible equivalent to tissue biopsy sampling. There is also the opportunity to monitor changes in tumor biology over time such as in response to therapy. Hence, the detection and isolation of CTCs may be a crucial tool to complement other biomarkers in order to personalize PDAC treatment.

## 3. Circulating Tumor Cells

**Figure 1:**
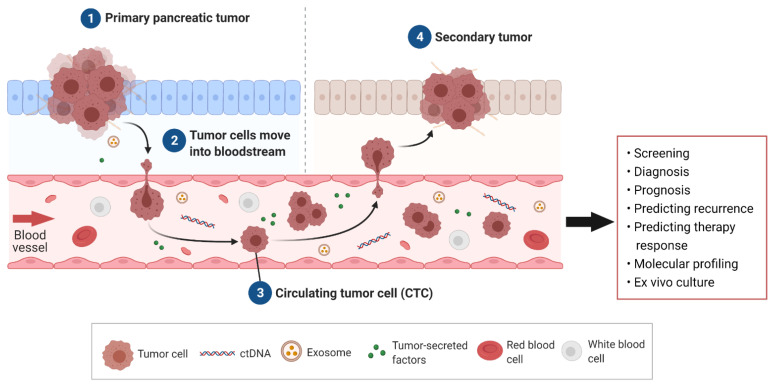
Circulating tumor cells (CTCs) arising in pancreatic ductal adenocarcinoma (PDAC) and their clinical utility. Tumor cells, along with other liquid biopsy biomarkers such as circulating tumor DNA (ctDNA), exosomes, and secreted factors such as metabolites, are secreted into the bloodstream from the PDAC tumor. Tumor cells, termed CTCs, travel through the blood to secondary sites to form metastases. Potential clinical applications of PDAC CTCs include screening, diagnosis, prognosis, predicting recurrence, and therapeutic response as well as insights into an individual’s tumor through molecular profiling and culture.

CTCs are malignant cells present in the bloodstream and are understood to be the “seeds” of metastasis. CTCs are shed from the primary tumor and travel through the vascular system to a secondary site, where given the correct environment, they will settle and multiply, thereby forming a metastatic tumor (Figure 1) [46]. CTCs have been studied extensively in breast, colorectal, and prostate cancer as a non-invasive assessment of disease progression and prognosis [47,48,49]. In PDAC, studies have largely focused on CTCs’ prognostic ability where the presence and number of CTCs has been correlated with worse survival [27,50].

### CTC Enrichment and Detection

**Figure 2:**
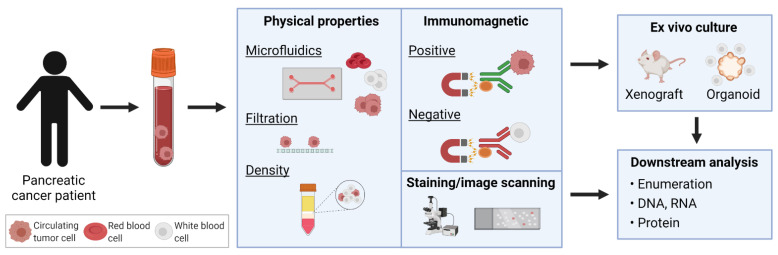
Isolation of circulating tumor cells (CTCs) in pancreatic cancer. Various enrichment and detection methods have been used to identify CTCs. Identified CTCs may be propagated by ex vivo culture through xenografts or organoids, or directly analyzed for enumeration and DNA/RNA/protein profiling.

CTCs were first discovered in 1869, when Australian physician, Thomas Ashworth, described cells similar to cancer cells from blood drawn from the saphenous vein [51]. However, only recent technological advancements have facilitated detection, enumeration, and isolation of these rare cells in a sensitive and reproducible manner for clinical and research applications. In PDAC, CTC counts have been found to be one of the lowest amongst solid cancers using the CellSearch^®^ CTC detection method [52]. Diverse techniques and technologies developed for CTC enrichment, isolation, and identification from peripheral blood samples have been thoroughly described [26,27,50,53]. Broadly, these methods employ strategies based on either the physical properties or surface phenotype of CTCs. These include size and density using filters or microfluidic devices, or detection using immuno-magnetic separation based on surface markers or high-resolution image scanning, in order to distinguish CTCs from erythrocytes and normal nucleated blood cells (Figure 2). 

Density centrifugation was employed in early studies to isolate CTCs based on size and density with generally low detection rates of 24% to 40% in PDAC patients [54,55,56]. Later studies used membrane filtration such as ISET (isolation by size of epithelial tumor cells) [57,58,59], MetaCell [60], and Screencell [40,61] provided much higher efficiencies from 66% to 96% detection amongst PDAC patients. Microfluidic devices, such as the NanoVelcro, have also displayed high detection efficiency of around 75% in treatment-naive patients [62,63]. Other microfluidic devices have reached higher than 80% detection rates [64,65]. Recently, SMART-Chip, an automated, modular microfluidic device incorporating impedance and imaging for rapid confirmation of the isolated cells, found CTCs in a PDAC patient as a proof-of-principal study [66]. Unfortunately, the limitation with this strategy of isolation, using their physical property, is that CTCs have been found to be equal or smaller in size to nucleated blood cells (ranging between 4 and 30µm) so specificity may be low [67,68]. 

The other common method of isolation is by immuno-magnetic separation via surface tumor antigens, also known as positive selection. The only FDA-approved method of CTC isolation thus far is CellSearch^®^ which utilizes immunomagnetic separation using epithelial markers on CTCs. It is the method which has been tested in the greatest number of studies but has performed relatively poorly with detection rates of 7–48% across various stages of PDAC (Table 1) [23,52,57,69,70,71]. A limitation of using epithelial markers is that they can be downregulated or lost as CTCs undergo epithelial-mesenchymal transition (EMT) [31,72]. Furthermore, CTCs are heterogeneous and to date, a universal CTC antigen remains elusive. Thus, another strategy of immunomagnetic separation is by negative selection where white blood cells are depleted. This increases the likelihood of isolating CTCs expressing various antigens however, some loss of CTCs has been noted while achieving 80% detection efficiency [73]. High resolution image scanning of CTCs is an enrichment-free method to identify CTCs. Fixed blood cells are spread onto slides and stained. CTCs are detected after slide images are analyzed. Two platforms that utilize this method include the RareCyte^®^ platform and Epic CTC platform, however they have, thus far, not been tested in PDAC patients [74,75]. 

A gold-standard CTC detection method remains elusive, although there are many emerging methods and technologies. One of the main reasons is the heterogeneous nature of CTCs. Each CTC detection technology exhibits merits and limitations, but these discrepancies make comparative studies difficult and consequently rarely undertaken. This has limited the molecular characterization of CTCs where the sub-population of CTCs that cause metastases, if it exists, is yet to be defined. The collection and analysis of CTCs from the portal vein is feasible in PDAC patients. In such cases, there were increased numbers of CTCs compared to peripheral blood [78,80,82]. This is consistent with expectations as the portal vein is the draining blood vessel from the pancreas and has not undergone hepatic filtration. However, it involves a more invasive procedure, limiting its applicability. Another consideration is blood collection as this is a crucial first step of the CTC detection workflow. The blood collection tubes used can impact on the number of CTCs detected [83,84]. A method that can reliably detect CTCs with high sensitivity and specificity as well as low user/site variability combined with the ability to undertake single-cell characterization would be useful in both the clinical and research settings.

## 4. Clinical Utility of CTCs in PDAC

### 4.1. Diagnosis and Early Detection

There is currently no established screening test for PDAC in the general population. Most patients with PDAC remain asymptomatic until late-stage disease at which point the tumor is large enough to cause biliary obstruction or invade adjacent nerves and cause pain [85]. PDAC is diagnosed with abdominal imaging (CT scan or MRI) and tissue diagnosis pathologically confirmed with a EUS-guided biopsy. Benign or premalignant pancreatic lesions (such as pancreatic intraepithelial neoplasms (PanINs), intraductal papillary mucinous neoplasms (IPMN), mucinous cystic neoplasms (MCN), and other cystic lesions) are a frequent incidental finding and are common in the general population (2.6% for >2 mm lesion) [86]. These are often detected during transabdominal imaging of asymptomatic individuals during investigation of unrelated symptoms or conditions. Surveying these pancreatic lesions with a high risk of malignant transformation, using a combination of CT, MRI, and EUS, is recommended to enable the early detection of PDAC [87]. Another subset of patients at increased risk of developing PDAC are “high risk individuals” that have a strong family history of PDAC or who carry genetic abnormalities that predisposition them to PDAC (Peutz Jeghers syndrome, STK11, p16/CDKN2A, BRCA1, BRCA2, ATM, PALB2, Lynch syndrome, and hereditary pancreatitis (PRSS1, PRSS2, and CTRC)). The Cancer of the Pancreas Screening (CAPS) program screens these patients through an annual EUS procedure [88,89]. Current screening and diagnosis involve complex imaging modalities and procedures and so a complementary or more accurate and easily accessible liquid biopsy test could drastically improve patient outcomes in PDAC. 

Considering metastasis is often thought of as occurring late in cancer progression, CTCs were not expected to be detected in premalignant and early stages of PDAC. However, a study in mice detected circulating epithelial cells (CECs) of pancreatic origin in the premalignant stages of a genetically-engineered pancreatic cancer mouse model [90]. This led to a pilot study by the same group in patients with IPMNs in which CECs were detected in 33% (7 of the 21) of IPMN patients without clinical diagnosis of cancer [91]. Studies have identified CECs in up to 88% of patients with precursor lesions, predominantly IPMN, undergoing surgical resection (Table 2) [92,93,94]. In one of the studies, CECs were found in all patients with high-grade dysplasia indicating its potential to stratify high-grade to low-grade IPMN and other benign cysts [93]. Thus, these pilot studies demonstrate that CECs can be detected in premalignant stages of PDAC, but larger confirmatory studies are required. There is also limited information concerning the phenotypic characterization of detected CECs and their propensity to “seed” at metastatic sites is unknown. Characterizing CECs could provide valuable insights into PDAC development and, if they are found to be the “seeds” of metastases, would shift the current paradigm of metastasis in PDAC. Due to the low number of circulating cells in the early stages of PDAC, technologies to extract and process large volumes of blood, such as leukapheresis, have being developed and would be ideal in this early stage setting but have not been validated [95].

CECs may identify patients earlier than conventional diagnosis as recently shown where the PanSeer assay, which detects ctDNA, was able to identify cancer patients 4 years prior to conventional diagnosis [98]. However, longitudinal CEC studies with adequate follow-up are currently lacking. Genetic studies with mathematical modeling in PDAC have suggested that the window of opportunity to diagnose and treat precursor lesions is almost 12 years, illustrating a potentially wide opportunity for early detection and preventative management [99]. Thus, screening for CECs could be incorporated to patients undergoing surveillance for benign or premalignant pancreatic lesions as well as “high risk individuals” in the CAPS program. Studies may be underway already where the current CAPS program, CAPS5 (NCT02000089), includes as part of their primary outcome, examination of CECs from pancreatic duct fluid. 

### 4.2. Prognosis and Recurrence

As with many other solid cancers [100,101,102,103], the detection and enumeration of CTCs has clinical utility as a prognostic marker in PDAC. Comprehensive reviews and meta-analyses of up to 19 studies comprising over 1300 PDAC patients have demonstrated that the detection of CTCs correlates with worse progression-free survival and overall survival [27,104,105,106]. This was observed despite the use of a variety of CTC detection methods, illustrating the strong prognostic value of CTCs. Further subset analysis of the studies illustrated both the robust principles underlying CTC hypothesis, and that the prognostic value of CTC enumeration held true for both Asian and Caucasian populations. 

Many studies have examined CTCs as a prognostic marker in resectable PDAC patients undergoing surgical resection [58,59,70,71,107,108,109,110]. CTC detection consistently correlates with early recurrence of disease. The CLUSTER study prospectively collected and evaluated CTCs using the ISET device longitudinally in one of the largest reported studies comprising over 200 patients with presumed PDAC [107]. Analysis of retrospective data found CTC enumeration preoperatively could predict early recurrence within 12 months after surgery in neoadjuvant-treated and first-line resected patients. Overall survival was significantly longer in the neoadjuvant-treated patients who lacked detectable CTCs [107]. Hence, CTCs could be used to stratify patients at the minimal residual disease setting following surgical resection who may have an increased risk of recurrence and benefit from earlier or more intensive treatments with investigational agents, although prospective randomized clinical trials are lacking.

Pre-operative CTCs could also be used to identify patients who are eligible for surgery. One study of 53 patients found that elevated pre-operative CTC counts (≥3 CTCs/4 mL using the NanoVelcro microfluidic device) was able to significantly distinguish between patients with occult metastatic disease and those with potentially curable, localized tumors with 85% sensitivity and 80% specificity [63]. 

A recent study examined circulating stromal cells, cancer-associated macrophage-like cells (CAMLs) in addition to CTCs in PDAC patients using CellSieve, a microfiltration device that isolates cells based on size exclusion [111]. Although 23% of PDAC patients had CTCs, 95% of patients had CAMLs. Both the number and size of CAMLs significantly correlated with advanced pathological stage and progression-free survival. No CAMLs were identified in the healthy control group. 

### 4.3. Chemotherapy Response

The ability of CTCs to predict response to chemotherapy in PDAC patients in the neoadjuvant and advanced settings is less clear. In a study with 57 patients undergoing surgery, those who had undergone neoadjuvant therapy had significantly lower median CTC counts compared to up-front chemo-naïve patients at time of surgery [107]. However, this pattern was not observed in a different study where no difference was observed in the 16 patients who had undergone neoadjuvant therapy [58]. There is difficulty in drawing firm conclusions from these studies as there are a number of biases such as patient selection, resectability criteria, and chemotherapy used. This is emphasized by the fact that preoperative clinical factors do not predict resectability in PDAC patients undergoing neoadjuvant chemotherapy [112]. 

One study showed that stable or decreased CTC numbers were detected in advanced patients with stable disease or who responded to chemotherapy [97]. This study used vimentin to enrich and detect mesenchymal CTCs using a microfluidic device in 100 PDAC patients. However, it should be noted that two patients with progressive disease also had low CTCs and minimal increases in CTCs over time. This illustrates low specificity and the bias of using a mesenchymal-only method for CTC detection. Another study found that the absence of CTCs (detected by CellSearch^®^) at 3 months after chemotherapy correlated with better overall survival [69]. In summary, it is difficult to conclude whether CTCs are able to predict chemotherapy response due to variations across studies with the use of different staging criteria, chemotherapy used, and method of CTC identification. Considering that the majority of PDAC patients are diagnosed with advanced disease and are given first line chemotherapy of either FOLFIRINOX or gemcitabine with nab-paclitaxel, CTCs could be used to identify which chemotherapy option would be best for the individual and, also, to identify a given patient’s eligibility to receive investigational therapies.

## 5. CTC Characterization

### 5.1. CTC Phenotype

The major advantage of CTCs in comparison to other liquid biopsy techniques is the detection of the whole tumor cell. This allows for the identification of other markers and characterization beyond the enumeration of CTCs. Most studies have focused on characterizing the metastatic ability of CTCs using cancer stem cell markers and mesenchymal markers, as it is thought that this will determine the subpopulation that is responsible for metastasis. Identifying this subpopulation would be clinically useful for monitoring patients and for targeting to prevent further cancer spread. However, such a CTC subpopulation remains elusive. Studies, thus far, have limited participant numbers and comparisons between studies is difficult. Often studies reach conflicting conclusions due to the CTC methods used resulting in the potential identification of different CTC phenotypes. A recently published experimental mouse model allowed for the examination of CTC dynamics such as shedding and propensity to metastasize, to enhance the characterization of CTCs [113].

The mesenchymal phenotype of CTCs correlates with disease progression in diverse cancers such as lung and breast cancer [114,115,116]. Conversely, an increased proportion of epithelial CTCs correlates with treatment response. This highlights the bias that may occur with epithelial- and mesenchymal-only detection methods and illustrates the need to phenotype and characterize CTCs rather than relying solely on their enumeration. This was shown in a recent study where CTCs were isolated using an epitope-independent approach involving microfluidics-based dielectrophoresis enrichment followed by a multiplexed immunostaining assay to identify epithelial, mesenchymal, partial-EMT (expressing both epithelial and mesenchymal markers), and stem cell-like CTCs [117]. The study found that total CTC counts did not correlate with any clinicopathological variables. However, there was a positive correlation between the proportion of partial-EMT cells and advanced disease, worse progression-free and overall survival, and earlier recurrence after resection. Another study also detected CTCs expressing both epithelial and mesenchymal markers in 36.4% of patients but in this case mesenchymal CTCs, and not the partial-EMT CTCs, significantly correlated to advanced stage and presence of distant metastasis [118].

Cancer stem cell markers have been examined to identify stem cell-like CTCs. Not only could these markers help identify the subpopulation of cells that cause metastasis, there is also evidence that the presence of these cells in the primary tissue correlates to resistance to treatment. For example, CD133, CXCR4, and ALDH1 expression has been shown to play a role in resistance to chemotherapy [119,120]. A number of stem cell markers have been identified in PDAC such as CD44, CD24, c-met, ALDH, CD133, and RORɣ, however, to date, CTC studies are limited to only one of these markers at a time [121]. Gene expression analysis of CTCs found that high expression of ALCAM, POU5F1B, and SMO were predictive of poor overall survival [41]. After chemotherapy, CTCs express higher expression of stemness and pluripotency genes (CD44, ALCAM, EPCAM, NOTCH1, POU5F1B, and PTCH1) or cancer stem cell drivers (VEGFB and STAT3). Examination of CTCs could also lead to the identification of novel drivers of metastases such as the *LIN28B* gene which was found to be prognostic [122]. Another study found that the cancer stem cell marker, CD133, helped identify a small subset of patients with progressive disease potentially illustrating progressive clonal treatment resistance [65]. However, a different study examining CD133+ CTCs did not find any predictive or prognostic value in the local or advanced disease settings [117]. 

CTC clusters, or circulating tumor microemboli, have been shown to have greater metastatic potential than single CTCs [123]. CTC clusters have a survival advantage in the circulation, protecting the tumor cells from apoptosis, shear stress, and immune response [124]. Neutrophils in breast cancer and platelets in PDAC has been found to chaperone CTC clusters [125,126]. As such, EpCAM-based methods such as CellSearch^®^ may exclude these CTC clusters based on their CD45 expression. CTC clusters have been detected in PDAC patients in several studies [41,117,125]. In one study, it was found that the presence of CTC clusters, rather than CTCs, was correlated with reduced overall survival, suggesting its use as a prognostic marker [127]. However, another study did not observe this, and a correlation was not observed between the presence of CTC clusters and patient survival [41]. CTC clusters could be a potential indicator of chemotherapy response [128]. 

### 5.2. CTC Genotype

Genotypic analysis of CTCs has been used to confirm the identity of CTCs in PDAC. This typically involves detecting the presence of the *KRAS* mutation which is found in close to 90% of PDAC patients [129]. 100% concordance for *KRAS* mutations in CTCs compared to their matched primary tissue was found in 5 PDAC patients [62], 19 PDAC patients [80], and 32 resectable PDAC patients [109]. However, a larger study with 58 patients did find discordance in 42% of patients where a different *KRAS* mutation was found compared to their primary tumor [130]. This could represent the natural evolution of the cancer where tumor cells acquired mutations in their secondary sites, heterogeneity within the primary tumor or technical limitations [131]. Hence, using only the *KRAS* mutation to confirm tumor identity with the primary tissue may not be sufficient, and may require the addition of other genetic mutations such as *TP53, SMAD4,* and/or *CDKN2A* which together account for the top four mutations observed in PDAC [129].

Further genotypic characterization of CTCs is currently constrained by the technical limitations of single-cell sequencing in single CTCs. A retrospective study using bulk CTCs found that an increase in *SMAD4* expression levels in CTCs was associated with longer progression free survival in advanced patients treated with gemcitabine/nab-paclitaxel [132]. A recent study utilized targeted single cell next-generation sequencing technology without pre-amplification to examine the three major driver genes: *KRAS, TP53,* and *SMAD4* in ISET-enriched single isolated CTCs [133]. In the future, single-cell sequencing may be more widely available for larger targeted genetic panels to determine specific therapies. A retrospective ctDNA study found 48% of 357 PDAC patients had a therapeutically relevant mutation [134]. 

Substantial progress has been made using ctDNA for targeted therapies. Some PCR-based single-gene and multigene assays, as well as high-throughput NGS-based multigene tests, have recently received FDA approval [135]. However, no routine test exists in PDAC yet. Although there is concordance between ctDNA and CTC mutations [136], further studies are required to examine if there is additional clinical benefit by examining the CTC genotype for targeted therapy. 

### 5.3. CTC Culture

Culturing CTCs will become essential for future drug discovery and targeting metastatic disease. While labor-intensive, this allows for the propagation of CTCs for downstream analysis thereby overcoming the limitations of limited cell numbers. Successful culture of CTCs has been accomplished in very few studies, mainly in prostate, breast, and lung cancer [137,138,139]. Initial studies used negative selection, namely RosetteSep^TM^, to deplete CD45-positive leukocytes, leaving untouched CTCs for ex vivo culture. This has been successfully achieved in 6 small-cell lung cancer patients using CTC-derived xenografts [140]. CTCs have also been isolated from the blood of prostate cancer patients and grown as organoids, which are 3D culture systems that self-organize to resemble the tissue of origin [141]. 

Current evidence indicates that PDAC CTC cultures are possible, however, further investigations are required to determine the correct conditions to establish CTC cultures reliably. One study investigated the use of extracellular matrix microarrays for isolating and culturing CTCs from mice engrafted with primary human PDAC tumors [142]. In another study, generated PDAC CTC organoids required co-culture with immune cells, but could only be propagated for up to 7 days [143]. A more recent study used a CTC organoid fibroblast co-culture system to examine cancer-induced stromal reprogramming of metabolic pathways [144]. Since organoid cultures have previously been generated from hard-to-obtain tissue samples (such as a tissue biopsy [145]), comparisons with matched CTC-derived organoids have not been reported. Such correlations would be clinically useful to determine if CTC-derived organoids could replace the need for tissue biopsies. Finally, CTC-derived organoids could facilitate precision/personalized approaches through drug screening, which are already underway in tissue-derived organoids [146]. 

## 6. Conclusions and Future Perspectives

Obtaining repeated tissue samples from PDAC patients is challenging due to the anatomical position of the pancreas whereby invasive procedures are required such as EUS-guided biopsies. Liquid biopsies are minimally invasive and can be easily collected over multiple time-points, enabling longitudinal, long-term disease surveillance and monitoring. Among the liquid biopsies, CTCs allow more in-depth characterization of tumor cells, including multiomic analysis and potential to culture for drug screening, possibly leading to personalized treatments. CTCs may provide a deeper understanding of an individual’s tumor characteristics, beyond the prognostic and diagnostic value which current small-scale studies have found in PDAC patients. However, the predictive value of CTCs is yet to be comprehensively investigated in the clinical setting. Large-scale prospective studies with CTC-guided management are needed to validate the clinical potential of CTCs. 

Considering that a “window of opportunity” from the earliest genetic alteration in a precursor lesion to the development of “full-blown” invasive cancer spans almost 12 years, future CTC studies should focus on screening and early detection of PDAC. CTCs have been detected in the early stages of PDAC, suggesting that metastasis may be an early event in PDAC. The early detection of PDAC could significantly improve the poor survival outcomes of PDAC when patients would be eligible for surgery and treatments are generally more effective. Screening using CTCs could be tested initially in “high risk individuals” before potentially extending this to the general population. 

Reliable and consistent CTC detection remains a challenge as current reports have adapted different CTC isolation techniques. Consequently, downstream phenotypic analysis may be biased and thereby hinder the identification of the subpopulation/s responsible for PDAC metastasis. Advances in single-cell technologies will allow for comprehensive characterization of CTCs, leading to insights into CTC biology, PDAC metastasis, and tumor heterogeneity. Although further investigation is required to optimize culture conditions, CTC-derived organoids could facilitate personalized medicine approaches. Ultimately, CTCs may provide a unique role in resolving important biological questions in PDAC evolution and clinical management.

## Figures and Tables

**Table 1:** Circulating tumor cell (CTC) detection using CellSearch^®^ in peripheral blood samples from patients with pancreatic ductal adenocarcinoma (PDAC).

Year of Report	Number of Patients	PDAC Stage	CTC Detection (% Patients)	Main Findings	Ref.
2004	16	Advanced	19	CTCs found in various cancers and negligible in healthy (0.3%)	[52]
2008	26	All	42	CTCs correlated with shorter survival	[76]
2011	54	Locally advanced and advanced	40	CTCs associated with shorter survival	[57]
2013	79	Locally advanced	11	CTCs correlated with poor tumor differentiation and shorter survival	[77]
2014	20	Local	20	No correlation between CTCs and survival. Portal vein CTCs (detected in 45%) associated with liver metastases	[78]
2015	45	All	20	CTCs mainly found in advanced disease. CTCs correlated with shorter survival	[79]
2015	14	All	21.4	Portal vein collection using EUS was feasible. Portal vein CTCs detected in 100% patients	[80]
2017	65	Advanced	32.3	CTCs correlated with presence of liver metastasis and shorter survival	[69]
2017	48	Advanced	48	No correlation between CTCs and survival. MUC1-positive CTCs correlated with shorter survival	[81]
2018	20	Local	25	MACS enrichment/Ariol vs CellSearch	[70]
2019	22	Local	32	CTC combined with GPC1-exosomes: 100% sensitivity and 80% specificity for diagnosis. CTC clusters with GPC1-exosomes correlated with shorter survival	[23]
2021	98	Local	7.1	CTCs correlated with recurrence and shorter survival	[71]

**Table 2:** Circulating epithelial cell (CEC) detection in benign or premalignant pancreatic lesions.

Year of Report	Number of Patients	CEC Method	CEC Method Type	CEC Detection Rates (% Patients)	Comments	Ref.
2014	Cystic lesion: 21 Healthy: 19	GEDI	Microfluidic	Cystic lesion: 33 Healthy: 0	Patients with cystic lesions and no clinical diagnosis of cancer (Sendai criteria negative)	[91]
2015	IPMN: 21 Healthy: 9	Screencell	Filtration device	IPMN: 49 Healthy: 0	CEC detection rates were similar in patients with benign (46%), premalignant (49%), and malignant (49%) lesions	[92]
2016	IPMN: 15 MCN: 2	NanoVelcro	Microfluidic	IPMN: 0 MCN: 0	Median CTC count in their PDAC cohort was 2	[62]
2017	IPMN: 16 MCN: 1 Healthy: 9	Screencell	Filtration device	IPMN: 75 MCN: 100 Healthy: 0	No morphologic differences between cells from different pancreatic diseases	[96]
2017	IPMN: 19 MCN: 1	ISET	Filtration device	IPMN: 58 MCN: 0	More likely found in patients with IPMNs with high-grade dysplasia	[93]
2018	IPMN: 20 Healthy: 11	CTC-iChip (followed by IF/RNA-ISH)	Microfluidic	IPMN: 88	Detected CECs likely from pancreatic lesions by RNA-seq	[94]
2018	IPMN: 13 MCN: 2 PanIN: 2	NanoVelcro	Microfluidic	IPMN: 0 MCN: 0 PanIN: 0	Median CTC count in the PDAC cohort was 2	[63]
2019	IPMN: 16 Healthy: 30	CytoQuest™ CR (Vimentin+)	Microfluidic	IPMN: 25 Healthy: 0	Using a cut-off value of 2 cells/4 mL of blood	[97]

IPMN: intraductal papillary mucinous neoplasms; MCN: mucinous cystic neoplasm; PanIN: pancreatic intraepithelial neoplasia; PDAC: pancreatic ductal adenocarcinoma; CEC: circulating epithelial cell; CTC: circulating tumor cell; GEDI: geometrically enhanced differential immunocapture; ISET: isolation by size of epithelial tumor cells; IF: immunofluorescence; RNA-ISH: RNA in situ hybridization.

## Data Availability

Data sharing is not applicable to this article. No new data were created or analyzed in this study.
